# Human Barometers

**Published:** 1888-08-04

**Authors:** 


### The Doctor's Pulpit.

### HUMAN BAROMETERS.

"One man in his life plays many parts." To not a few it is given to act as very efficient barometers. There are people who could gauge the weather with considerable correctness, even if locked up in a darkened room. The human nervous system is a strange and to a large extent inexplicable study. The anatomist has his view of it, and sometimes a miserably wooden and inadequate view it is. The metaphysician approaches the subject from another side, but we are bound to admit that, taking metaphysicians and anatomists in the mass, the former have, on the whole, given a much more full and reasonable account of the use and value of that central portion of the nervous system called the brain than the latter. The anatomists will laugh this idea to scorn. Do they not saw through the skull and take out the brain with their own hands? Do they not inspect and examine and name and appraise its hemispheres, its fissures, its cerebrum and its cerebellum, its pons varolii and its medulla oblongata? Are not its ventricles familiar to the handles of their scalpels? Do they not know its commissures, the ramifications of its fibres, its centres large and small, the origins of its nerves, and the use and function of almost every part? Most true; and so does the professor of agriculture know about roots, and manures, and stockbreeding, and silos, and everything else under the sun. But when he takes a farm of his own he often lands himself in the bankruptcy court, whilst Hodge, who merely uses the methods of common sense, common observation, and common experience, slowly but surely produces good crops and fine cattle, and builds up at length a fair sum in the three per cents.

There is still a third class who study the human nervous system. They are a somewhat mixed assemblage. Among them may be included mesmerists, electrobiologists, spiritualists, table-turners, faith-healers, interpreters of dreams, and other such-like persons. Some of these are perfectly honest people; others, no doubt, are charlatans pure and simple; and not a few belong to that numerous class whose intentions are good enough, but whose judgment and will are so feeble that they easily yield themselves up to the uncontrolled influence of others.

Now all these odd people are worthy of attention and study. Many of them have a substratum of solid truth and of undeniable experience to go upon. Nothing can be more contrary to the finest scientific temper than to brush them all aside as idiots or cheats, as the materialist almost invariably does, unless it be the lofty contempt with which they are regarded by some of the superior persons among the metaphysicians. The fact that requires more constant recognition by everybody is that the possible functions of the human brain in their fullest extent are not, at any rate yet, perfectly known and precisely defined. The brain has for its servants the eyes, the ears, the nose, the tongue, the limbs, and every part of the body which is subject to the control of voluntary muscles. But is its activity limited to operations subserved by these various parts? Most assuredly not. Besides, although there is a general similarity between different human brains, the divergences are often wide as the poles asunder. Whilst one man is a Mendelssohn and fills the world with his fame as a musician, another cannot get through a single bar correctly. Here is a senior wrangler, who may be the Newton of the future; there is his companion, who, even yet, can hardly understand why the square of the hypothenuse of a right-angled triangle should be equal to the squares of the other two sides.

We plead then for, and insist upon, the recognition, in popular thought as well as in medical practice, of an egoistic, personal, nervous factor, a peculiarity, a real, actual, and effective something which may influence men and women in all kinds of unexpected ways, which may make or mar their whole social, or even their moral life. A patient sits before his doctor and complains of a strange and unbearable feeling in the head; his ears vibrate, he says; his brain tingles, his spirits are depressed, he feels so limp that he could fall prone on the ground. What is the matter with him? he wants to know. He was better yesterday; to-day he is worse. At the present moment he feels as if he could commit suicide. If, now, both patient and doctor will look out of the window, they will probably see the whole sky black and heavy with summer storm-clouds. The air is charged with electricity; the birds are hushed; the cattle stand still in the fields; the very leaves of the trees cease to move to and fro, and tremble as if in fear. What is the nexus between the man's brain and the black and surcharged sky? What is it that makes him for the moment as miserable as a lost soul? Put him into an express train, and take him to a hilly and bracing region fifty miles beyond the outer ranges of the storm. What is the result? He is now as joyous as he was before wretched. All his symptoms have disappeared. He is perfect. But in his wellbeing he is just as much a barometer as in his misery. In each case it is the atmospheric condition which accounts for the symptoms. The only difference is that the latter state, being joyous and full of the sense of wellbeing, is not noticed; whilst the former, being as it is the very inferno of utter misery insists upon being recognised and taken account of. This is one illustration of facts and circumstances familiar to thousands.

Not only are many human beings barometers so far as susceptibility to atmospheric conditions is concerned, but they are most sensitive indicators of all kinds of happy and unhappy, adverse and favourable circumstances. Men and women are not mere wooden or steel mechanisms; nor yet are they ordered assemblages of cunningly-compounded chemical constituents. Over and above everything the mechanical or the chemical philosopher can take account of is a conscious ego which works through and by means of the nervous system, and which the nervous system exalts to rapture or sinks to despair, according as it is influenced by external circumstances. The conscious ego ought to be, and largely is, the master of the nervous system. But just as often it is, if not exactly the slave, at least the very much tried and afflicted companion. Thousands of men and women have fervently wished they had no nerves. So far as our experience instructs us there is no intellectual life without n.rves. In other spheres than this intellectual activity may be possible under other conditions. For us the ego works by means of the brain, and by that means only.

Many physicians and other persons despise the nervous factor and the consciousness which is so much influenced by its variations. Such a view of life does not prove their superior capacity, but rather the opposite. It is a refusal to take account of the facts of the case. The Frenchman,—or was it T. P. Barnum?—when told the facts were against him, exclaimed, "So much the worse for the facts." That was amusing, but it was not practical. Facts must be taken into account; they insist upon being recognised and dealt with. The man who lays his plans and resolves to carry them through with or against facts, proves himself in the long run not merely a rash fool, but a failure. The only really able man is he who sees the facts, admits them, and works with them instead of against them. The man who thus sees clearly all the circumstances, and works with them, is like a skilful knight carving his way to fortune with a sharp sword. He who shuts his eyes and resolves to conquer without first seeing what it is he has to do battle against is no knight at all, but a mere untutored savage trying to fight the battles of civilised life with a wooden club. The writer has left himself no space for dealing with the barometrical question of how to modify and improve an unduly sensitive nervous system. The subject is one of great importance to a large and suffering class; and if opportunity offers it will be returned to at some future time.

